# LncARSR sponges miR-129-5p to promote proliferation and metastasis of bladder cancer cells through increasing SOX4 expression

**DOI:** 10.7150/ijbs.39461

**Published:** 2020-01-01

**Authors:** Chunxian Liao, Zhaolin Long, Xinji Zhang, Jianli Cheng, Fuming Qi, Shihao Wu, Tao Huang

**Affiliations:** 1 Department of Urology, Shunde Hospital, Southern Medical University (The First People's Hospital of Shunde), Foshan City 528308, Guangdong Province, China; 2 Institute of Urology, Peking University Shenzhen Hospital, Shenzhen 518000, China

**Keywords:** Bladder cancer, lncARSR, miR-129-5p, ceRNA, SOX4

## Abstract

Emerging evidences have indicated that long non-coding RNAs (lncRNAs) are potential biomarkers, playing important roles in the development of cancer. LncRNA Activated in RCC with Sunitinib Resistance (lncARSR) is a novel lncRNA that functions as a potential biomarker and is involved in the progression of cancers. However, the clinical significance and molecular mechanism of lncARSR in bladder cancer (Bca) remains unknow. In this study, we discovered that lncARSR was significantly up-regulated in bladder cancer. In addition, increased expression of lncARSR was positively correlated with higher histological grade and larger tumor size. Further experiments demonstrated that suppression of lncARSR attenuated the proliferation, migration, invasion and epithelial-mesenchymal transition (EMT) process of Bca cells. Mechanistically, lncARSR was mainly located in the cytoplasm and acted as a miRNA sponge to positively modulate the expression of Sex-determining region Y-related high-mobility-group box transcription factor 4 (SOX4) via sponging miR-129-5p and subsequently promoted the proliferation and metastasis of Bca cells, thus playing an oncogenic role in Bca pathogenesis. In conclusion, our study indicated that lncARSR plays a critical regulatory role in Bca cells and lncARSR may serve as a potential diagnostic biomarker and therapeutic target for bladder cancer.

## Introduction

Bca is the most common tumor of urinary system, with high incidence and mortality. It is estimated that approximately 549,000 new cases and 200,000 deaths in the worldwide in 2018 1. Despite great improvements in Bca treatments have been made in the past decades, such as surgery, chemotherapy and immunotherapy, the 5-year survival rate and prognosis of those patients is still in a dilemma 2, 3. Early diagnosis and treatment of this disease is closely related to the prognosis of patients 4. Recurrence and metastasis of Bca is the main reason for the failure of current therapy 5. However, precise molecular biological changes in Bca remain unclear. Therefore, it is urgent to identify novel biological targets in the diagnosis and treatment of Bca.

Long non-coding RNA (lncRNA), a novel type of RNA molecules, is over 200nt in length. Owing to lack of open reading frame (ORF), lncRNAs are not able to encode the protein 6, 7. Recently, it is reported that lncRNAs play important roles in cellular functions, such as cell proliferation, apoptosis, invasion, gene regulation and chromatin remodeling 8. Moreover, large proportions of lncRNAs are involved in the cancer progression and metastasis 9, 10. The expression of lncRNAs is obviously tissue and cell type specific. They are highly dysregulated in various tumors, serving as oncogenes or tumor suppressors. In many cases, lncRNAs act as guides or scaffolds to modulate protein to DNA or protein to protein interactions, as enhancers to control gene expression, as microRNA(miRNA) sponge to capture miRNAs 11, 12. In addition, lncRNA can act as readouts of active cellular programs or signal conductors of specific cellular states, could detect the pathology of cancers and provide prognostic value for cancer patients 13, 14. For instance, lncRNA MALAT-1 is overexpressed in various cancers, and high expression of MALAT-1 is obviously associated with poorer overall survival and disease-free survival in gastrointestinal carcinomas 15. LncRNA HOTAIR is augmented in the primary and distant metastatic breast cancer, predicting poor prognosis of cancer patients. It can also enhance the invasion and metastasis of breast cancer cells through inducing PRC2 retargeting and modulating the H3K27 methylation 16. LncRNA HOXD-AS1 can promote glioma cell migration and invasion through serving as miRNA sponge. It competitively binds to miR-130a to up-regulate the transcriptional factor E2F8 expression 17. Therefore, a comprehensive genomic characterization of lncRNA alterations across the cancer progression and metastasis is urgently needed and may contribute to the identify novel diagnostic and therapeutic methods for cancer.

LncRNA Activated in Renal cell carcinoma with Sunitinib Resistance (lncARSR) is a novel identified lncRNA containing four exons with 591 nt in length and located on chromosome 9. Recently, lncARSR was regarded as a potential biomarker and got involved in the progression of different cancers. It is suggested that lncARSR acts an oncogene and plays a critical role in the development of renal cancer, hepatocellular carcinoma and ovarian cancer 18-20. However, detailed mechanism induced by lncARSR participates in tumorigenesis remains to be clarified. Moreover, the biological function and underlying mechanism of lncARSR in the development of Bca are remains unknow.

In our study, we presented that lncARSR was significantly up-regulated in Bca tissues compared with corresponding non-tumor tissues in total of 62 Bca patients. High expression of lncARSR was significantly associated with higher histological grade and larger tumor size. Moreover, attenuated expression of lncARSR inhibited the proliferation, migration and invasion of Bca cells *in vitro* and vivo. Mechanistically, lncARSR acted as a nature miRNA sponge to positively control SOX4 expression by sponging miR-129-5p in a ceRNA-dependent manner. Suppression of miR-129-5p could reverse the malignant phenotype inhibition of Bca cells induced by silencing lncARSR. Hence, our study revealed that lncARSR may become a powerful tumor biomarker for the diagnosis and treatment of Bca.

## Material and methods

### Clinical samples collection

Between 2012 and 2017, 62 Bca patients who had received radical cystectomy were included in this study. Fresh Bca tissue and matched normal tissues were snap-frozen in liquid nitrogen immediately after resection. All patients included in this study have signed the informed consent. The study was approved by the institutional research ethics committee of Shunde Hospital.

### Cell culture

All the cell lines included in this study were obtained from the Institute of Cell Biology, Chinese Academy of Sciences (Shanghai, China). The RT4 and 5637 cells were cultured in RPMI-1640 Medium (Invitrogen, Carlsbad, CA, USA). The SW780, J82, TCCSUP, UMUC-3 and T24 cells were grown in the DMEM medium (Invitrogen, Carlsbad, CA, USA). The SV-HUC1 cells were grown in F12K medium (Invitrogen, Carlsbad, CA, USA). All the medium was mixed with 10% fetal bovine serum (FBS) and 1% antibiotics. Cells were cultured in a humidified incubator with an atmosphere of 5% CO2 at 37 °C.

### shRNAs and anti-miRNA inhibitors

Two shRNAs targeting lncARSR (lncARSR-1,2) and negative control shRNA (shRNA-NC) were purchased from GenePharma (Suzhou, China). The shRNAs sequences for lncARSR were 5'-GCATGAAGAACTCCAACTTCA-3' and 5'-GAGCATGAAGAACTCCAACTT-3'. Anti-miR-129-5p inhibitor (anti-129-5p) and anti-miR negative control (anti-NC) were ordered from RiboBio Corporation (Guangzhou, China). J82 and 5637 cells were seeded in 6-well plates 20 hours (h) prior to miRNA inhibitor or shRNAs transfection with 50-70% confluence. Cell transfection was performed by Lipofectamine 3000 (Invitrogen, Carlsbad, CA, USA) according to the manufacturer's instructions. Stable cell lines were selected by 300µg/ml of neomycin for 2 weeks.

### RNA extraction and quantitative real-time PCR

Total RNA from the tissues and transfected cells were extracted by using the Trizol regant (Invitrogen, Carlsbad, CA, USA). Complementary DNA was synthesized with a reverse transcription kit (Takara Biomedical Technology, Dalian, China). Quantitative real-time PCR (qRT-PCR) was excuted by ABI PRISM 7500 Fluorescent Quantitative PCR System (Applied Biosystems, Foster City, CA, USA). The primer sequences included in this study were shown in Supplementary Table 1. GAPDH or U6 small nuclear RNA were chosen as the internal control. Experiments were repeated at least three times.

### Western blotting analysis

Cells were lysed by utilizing RIPA reagent (Beyotime, Beijing, China) mixed with protease inhibitor cocktail. Total protein was separated by 0% SDS-PAGE gels electrophoresis and transferred to PVDF membranes. The membrance was blocked with Tris-buffered saline (TBS) containing 5% nonfat milk for 1h and incubated with primary antibodies at 4 °C overnight. Autoradiograms were analyzed by densitometry by using Quantity One software (Bio-Rad). GAPDH was served as a control and antibodies against SOX4, E-cadherin, N-cadherin and Snail were purchased from Cell Signaling Technology (Danvers, MA, USA).

### Cell proliferation assay

Cell Counting Kit-8 (CCK-8) assay, colony-formation assay and 5-ethynyl-20-deoxyuridine (Edu) assay were used to detect cell proliferation. For CCK-8 assay, the absorbance of each well was determined by a microplate reader (Bio-Rad, Hercules, CA, USA) at 450nm. Edu assay was performed by using an EdU Apollo DNA *in vitro* kit (RIBOBIO, Guangzhou, China) according to the manufacturer's instructions. The image of Edu assay was observed under a 20X objective. Experiments were repeated at least three times.

### Wound healing assay

Cell migration was determined by wound healing assay. The transfected cells were seeded in 6-well plates at 48 h post transfection. A yellow pipette tip was used to scrach a wound field when cells grow to 90% confluent. The migrated cells were monitored with a digital camera system after 24 h. Experiments were repeated at least three times.

### Cell invasion assay

Cell invasion was determined by a transwell insert (8μm, Corning). 24 h after transfection, 1× 10^5^ cells were seeded in the upper chamber coated with Matrigel (BD Bioscience). 500µl of corresponding medium containing 10% FBS was added to the lower chamber. The invasive cells were stained with 0.1% crystal violet and photographed. At last, the stained cells were washed by 33% of glacial acetic acid. The absorbance was measured by a microplate reader at a wavelength of 570 nm. Experiments were repeated at least three times.

### RNA pull-down assay

To pull down the miRNA by lncARSR, J82 and 5637 cells were transfected with Biotin-labeled lncARSR. For overnight, the pull-down products were treated with RNase-free DNase I and RNeasy Mini Kit (QIAGEN, Germany). The bound miRNAs in the pull-down products was extracted, reversed and quantified by qRT-PCR. Experiments were repeated at least three times.

### Dual-luciferase reporter assay

The vectors of lncARSR-Wild Type (WT) or Mutant (Mut) were constructed and transfected into Bca cells along with miR-129-5p mimics or negative control. The vectors of SOX4-WT or MT were constructed and transfected into Bca cells along with miR-129-5p mimics or negative control. At 48 h post transfection, luciferase activity was measured using the Dual-Luciferase Reporter Assay System (Promega). Experiments were repeated at least three times.

### Tumor xenografts

The tumor xenograft assay was approved by the institutional research ethics committee of Shunde Hospital. The mice were raised in strict according to the care and use of laboratory animals. 5-week old male nude mice were randomly divided into the shlncARSR group and shctrl group (n=4 for each group). Approximately 5× 10^6^ 5637 cells stably transfected with shlncARSR plasmids or control vector were subcutaneously injected into the upper back of BALB/c-Nude mice. The mice were sacrificed after 6 weeks.

### Statistical analyses

All experimental data from three independent experiments were represented as mean±standard deviation (SD). Data analysis was executed using SPSS 19.0 (SPSS, Chicago, USA). Group difference was analyzed by Student's t test or χ2 test. P<0.05 was considered as statistically significant.

## Results

### LncARSR is up-regulated in Bca tissues and cell lines

**Figure 1:**
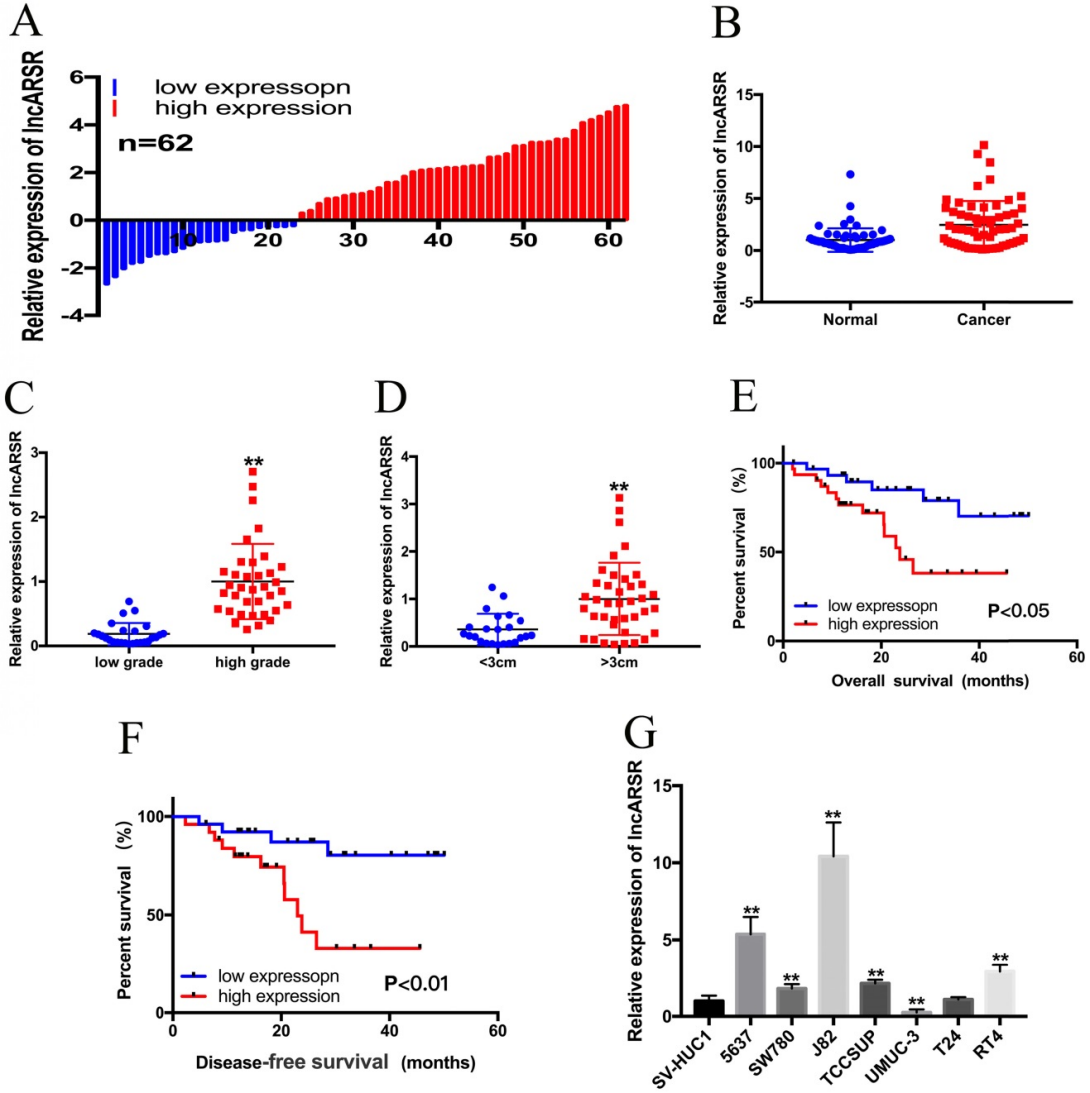
** The expression of lncARSR in Bca tissues and cell lines. (A)** 62 Bca patients were included in this study. **(B)** Relative expression of lncARSR in paired Bca tissues and normal tissues were shown. **(C)** LncARSR is up-regulated in Bca patients with higher histological grade. **(D)** LncARSR is up-regulated in Bca patients with larger tumor size. **(E** and **F)** Kaplan-Meier plots of the overall survival and disease-free survival of Bca patients with high and low expression of lncARSR. **(G)** LncARSR is up-regulated in Bca cell lines compared to that in SV-HUC1 cell. Data are shown as mean ± SD. *p < 0.05; **p < 0.01.

qRT-PCR assay was performed to detect the relative expression level of lncARSR in Bca tissues and cell lines. LncARSR was up-regulated in 63% (39/62) of cancer tissues compared with normal tissues (Figure. 1A, B). In addition, increased lncARSR expression was positively corelated with higher histological grade (Figure. 1C) and larger tumor size (Figure. 1D). The correlations between lncARSR expression and clinicopathological features of patients are shown in Table 1. Kaplan-Meier survival curve revealed that patients with high lncARSR expression showed a reduced overall survival time (Figure. 1E) and recurrence-free survival time (Figure. 1F). High levels of lncARSR were also detected in 5637, J82, SW780, TCCSUP and RT4 (Figure. 1G). J82 and 5637 cells were chosen for further experiments.

### Knockdown of lncARSR suppress the proliferation, migration and invasion of Bca cells

**Figure 2:**
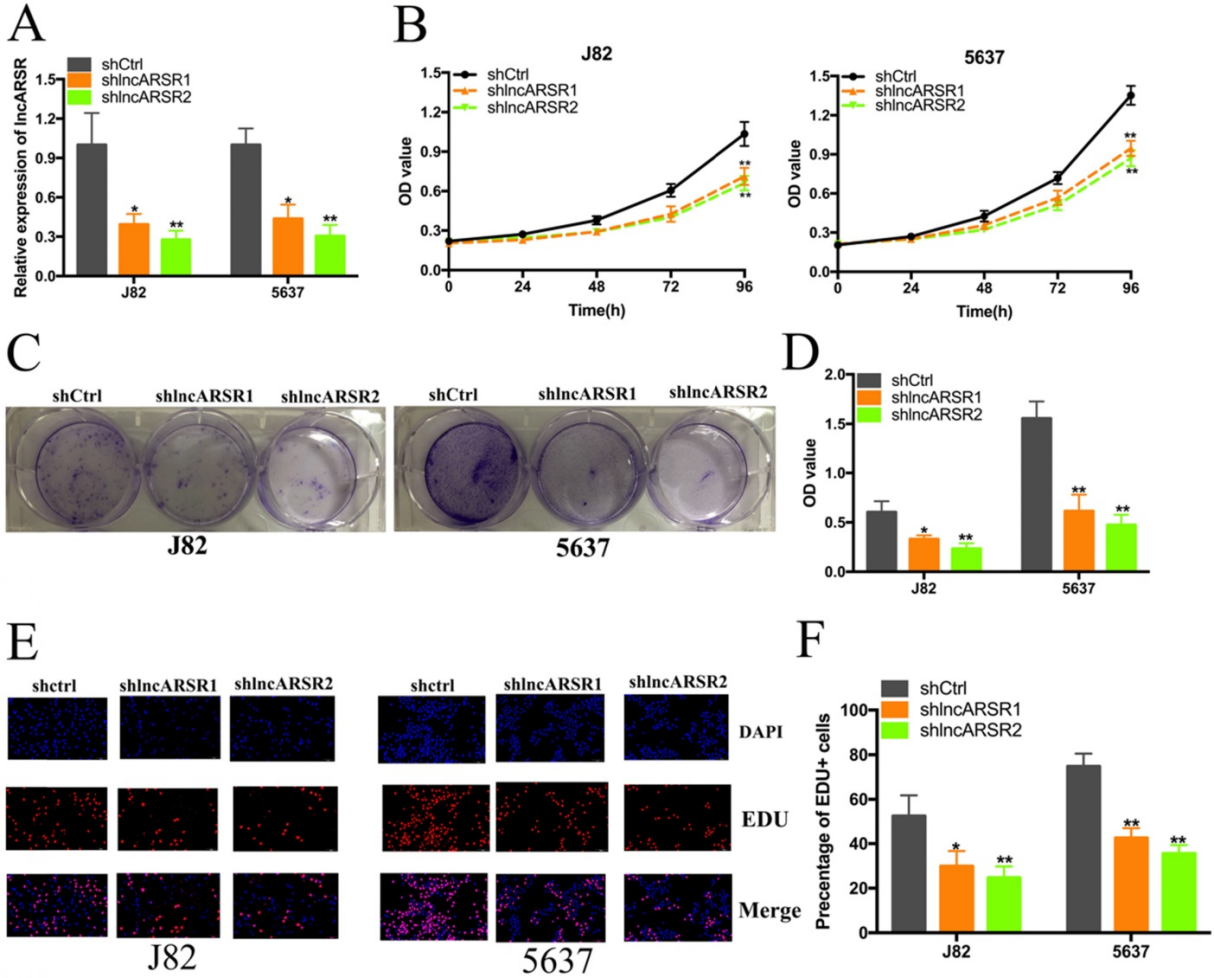
** Knockdown of lncARSR inhibited cell proliferation. (A**) LncARSR expression was suppressed by shlncARSR. **(B**) The growth cure changes of Bca cells were determined using CCK-8 assay. **(C** and **D)** The cell proliferation changes of J82 and 5637 cells were determined using colony-formation assay. (E and F) EDU assay showed that suppression of lncARSR attenuated proliferation of J82 and 5637 cells.

**Figure 3:**
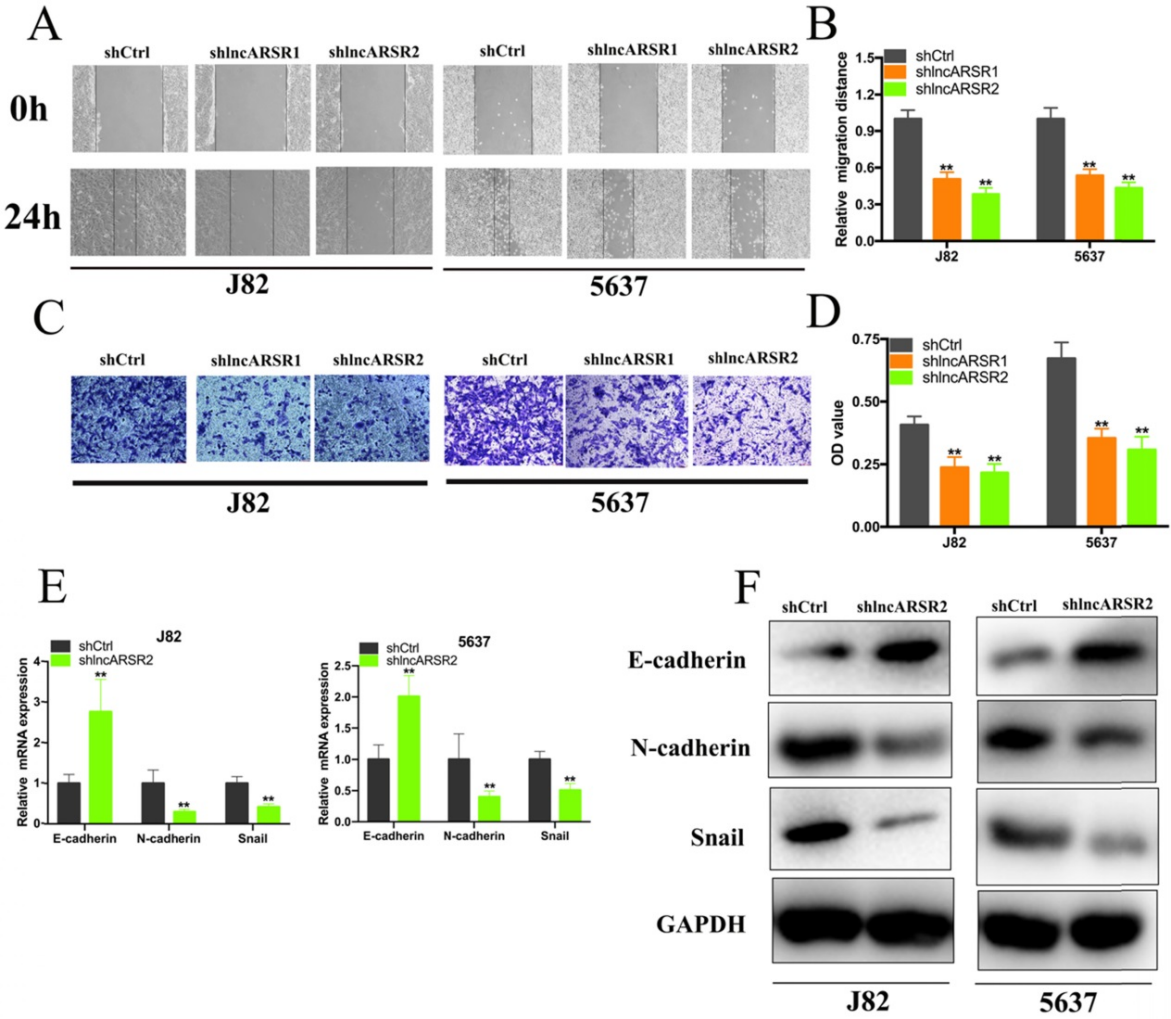
** Knockdown of lncARSR inhibited cell migration, invasion and EMT process. (A** and** B)** Wound healing assay showed that inhibited cell migration by silencing lncARSR was observed in Bca cells. **(C** and **D)** Transwell assay presented that the invasive abilities of bladder cancer cells were decreased by silencing lncARSR. **(E** and** F)** Knockdown of lncARSR increased the expression of E-cadherin and decreased the expression of N-cadherin and vimentin in bladder cancer cells. Data are shown as mean ± SD. *p < 0.05; **p < 0.01.

Given that lncARSR is up-regulated in Bca tissues and cell lines, we guessed that lncARSR could affect the biological activity of Bca cells. Two shRNAs targeting the coding region of lncARSR were used to suppress the expression of lncARSR (Figure. 2A). Cell proliferation ability was determined by performing CCK-8 assay, colony-formation assays and Edu assay. CCK-8 assay demonstrated that suppression of lncARSR slowed down the growth curve of J82 and 5637 cells (Figure. 2B, C). Inhibited cell proliferation was obviously observed in J82 and 5637 cells by silencing lncARSR (Figure. 2C-F). Wound healing assay showed that cells transfected with shRNA-lncARSR (shlncARSR) suffered a slower closing of scratch wounds compared with the negative control group (Figure. 3A, B). Transwell assay showed that of knockdown of lncARSR significantly the invasion of J82 and 5637 cells (Figure. 3C, D). We further investigated whether lncARSR regulated EMT process of Bca cells. qRT-PCR and western blotting assay were performed to detect the expression of EMT markers. As shown in Figure. 3E, F, knockdown of lncARSR increased E-cadherin expression and suppressed the expression of N-cadherin and Snail in Bca cells. These results suggested that knockdown of lncARSR inhibited the proliferation, migration and invasion of Bca cells.

### LncARSR directly interacts with miR-129-5p

**Figure 4:**
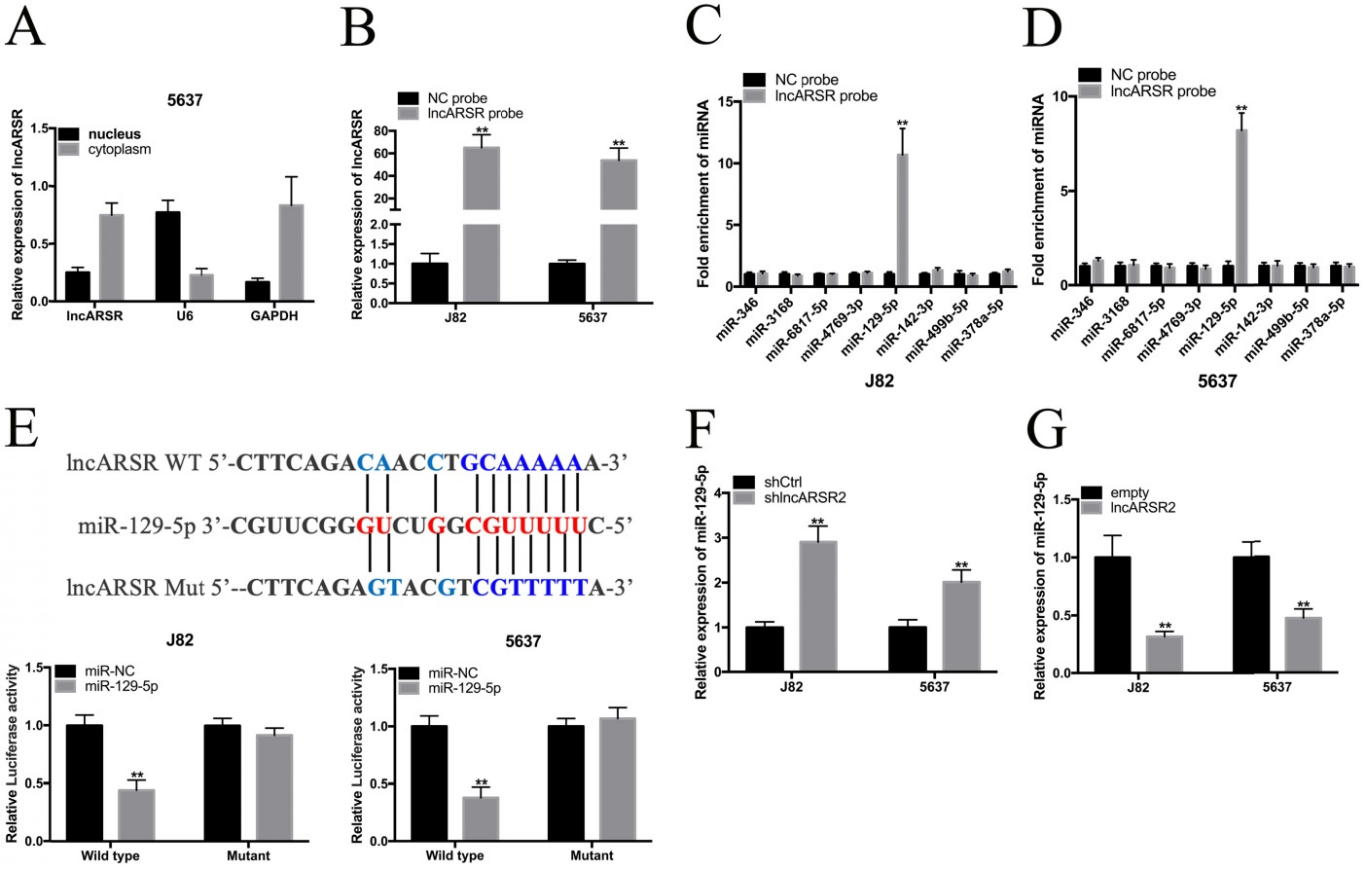
** LncARSR directly interacted with miR-129-5p. (A)** LncARSR was mainly distributed in the cytoplasm of 5637 cell. **(B)** Detection of lncARSR using qRT-PCR in the sample pulled down by biotinylated lncARSR and negative control (NC) probe. **(C** and** D)** Detections of miR-346, miR-3168, miR-6817-5p, miR-4769-3p, miR-129-5p, miR-142-3p, miR-499b-5p and miR-378a-5p using qRT-PCR in the J82 and 5637 sample pulled down by biotinylated lncARSR and NC probe. **(E)** Bioinformatics analysis showed that the sequence of lncARSR is complementary to the seed sequence of miR-129-5p. Dual-luciferase reporter assay showed that lncARSR-Wt and miR-129-5p mimic co-transfection significantly inhibited luciferase activity. **(F** and** G)** The expression levels of miR-129-5p in J82 and 5637 cells transfected with lncARSR shRNA or pcDNA-3.1 lncARSR were evaluated by qRT-PCR. Data are shown as mean ± SD. *p < 0.05; **p < 0.01.

The biological function and molecular role of lncRNAs is closely associated its subcellular localization. By using RNA-FISH assay, we detected the subcellular localization of lncARSR. As shown in Figure. 4A and B, lncARSR was predominantly located in the cell cytoplasm. Hence, lncARSR may severe as a natural miRNA sponge to regulate the expression of target genes. Through using the miRDB database, we observed that lncARSR had putative binding sites with miR-346, miR-3168, miR-6817-5p, miR-4769-3p, miR-129-5p, miR-142-3p, miR-499b-5p and miR-378a-5p. Then, we applied the biotin-labeled pull down system to detect the specific miRNAs that lncARSR could directly interact with. We found that a significant amount of miR-129-5p in the lncARSR pulled down pellet compared with the negative control group by performing qRT-PCR assay, but the amount of miR-346, miR-3168, miR-6817-5p, miR-4769-3p, miR-142-3p, miR-499b-5p and miR-378a-5p in the lncARSR pulled down pellet had no significant increase compared with negative control group. Dual-luciferase reporter assay showed that lncARSR-WT and miR-129-5p mimics co-transfection significantly suppressed the luciferase activity while lncARSR-MT and miR-129-5p mimics co-transfection had no effects on the luciferase activity (Figure. 4E). In addition, we found that knockdown of lncARSR increased miR-129-5p expression while overexpression of lncARSR decreased the expression of miR-129-5p (Figure. 4F, G).

### Suppressing of miR-129-5p reverses malignant phenotypes inhibition of J82 and 5637 cells induced by silencing lncARSR

**Figure 5:**
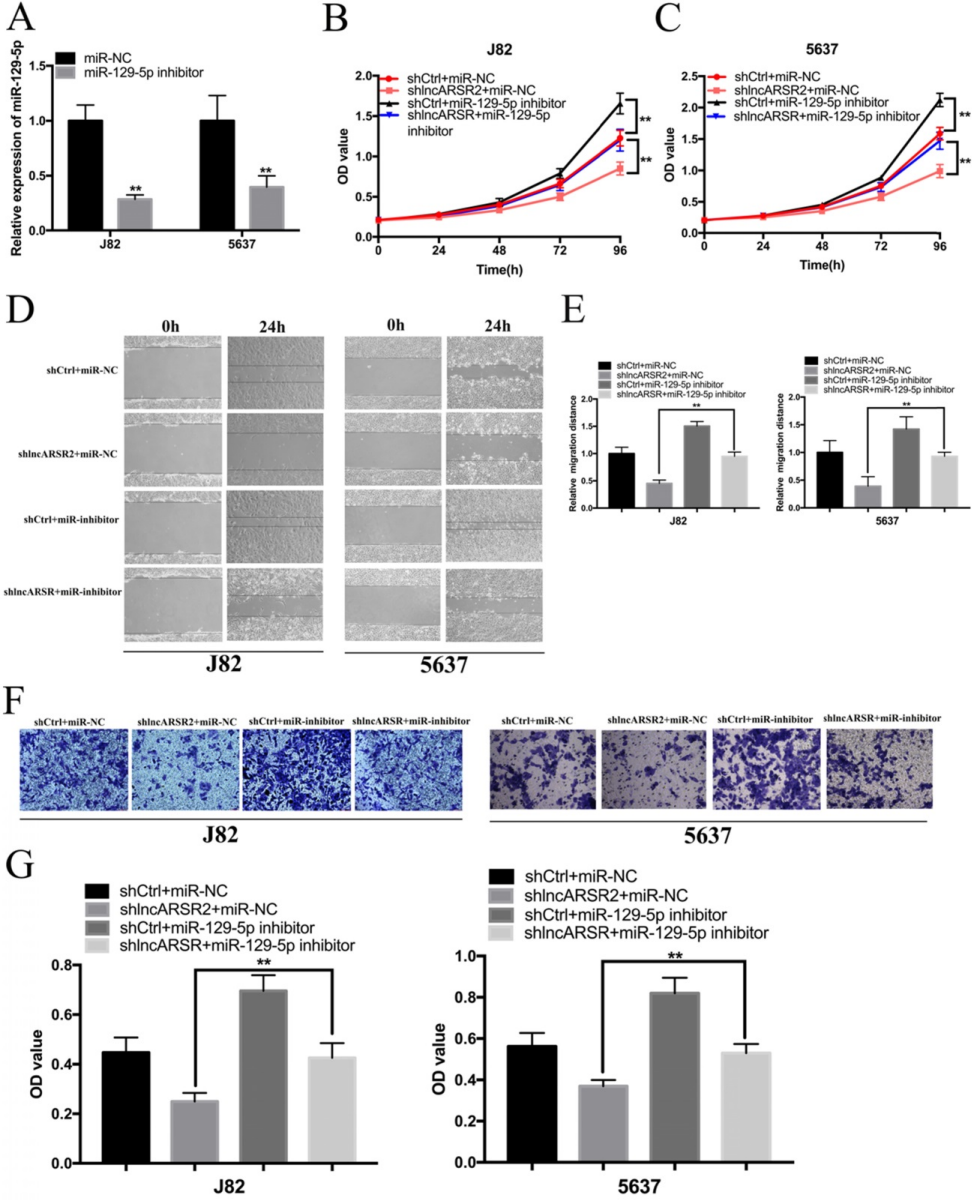
** Suppression of miR-129-5p reversed malignant phenotypes inhibition of bladder cancer cells induced by silencing lncARSR. (A)** The miR-129-5p inhibitor inhibited the expression of miR-129-5p in Bca cells. **(B** and** C)** The miR-129-5p inhibitor significantly reversed cell proliferation inhibition induced by silencing lncARSR. **(D** and **E)** The miR-129-5p inhibitor significantly reversed cell migration inhibition induced by silencing lncARSR. **(F** and** G)** The miR-129-5p inhibitor significantly reversed cell invasion inhibition induced by silencing lncARSR. Data are shown as mean ± SD. *p < 0.05; **p < 0.01.

We further investigated whether lncARSR regulated malignant phenotypes of J82 and 5637 cells via miR-129-5p-dependent manner. miR-129-5p inhibitor could significantly suppress the expression of miR-129-5p in Bca cells (Figure. 5A). CCK-8 assay showed that miR-129-5p inhibitor significantly reversed cell proliferation inhibition of Bca cells induced by silencing lncARSR (Figure. 5B, C). Wound healing assay suggested that suppression of miR-129-5p significantly reversed cell migration inhibition of Bca cells induced by lncARSR repression (Figure. 5D, E). Transwell assay showed that knockdown of miR-129-5p significantly reversed cell invasion of Bca cells caused by silencing lncARSR (Figure. 5F, G). These results suggested that lncARSR promotes malignant phenotypes of Bca cells via miR-129-5p-dependent manner and miR-129-5p may severe as a tumor suppressor in the Bca cells.

### MiR-129-5p directly target SOX4

**Figure 6:**
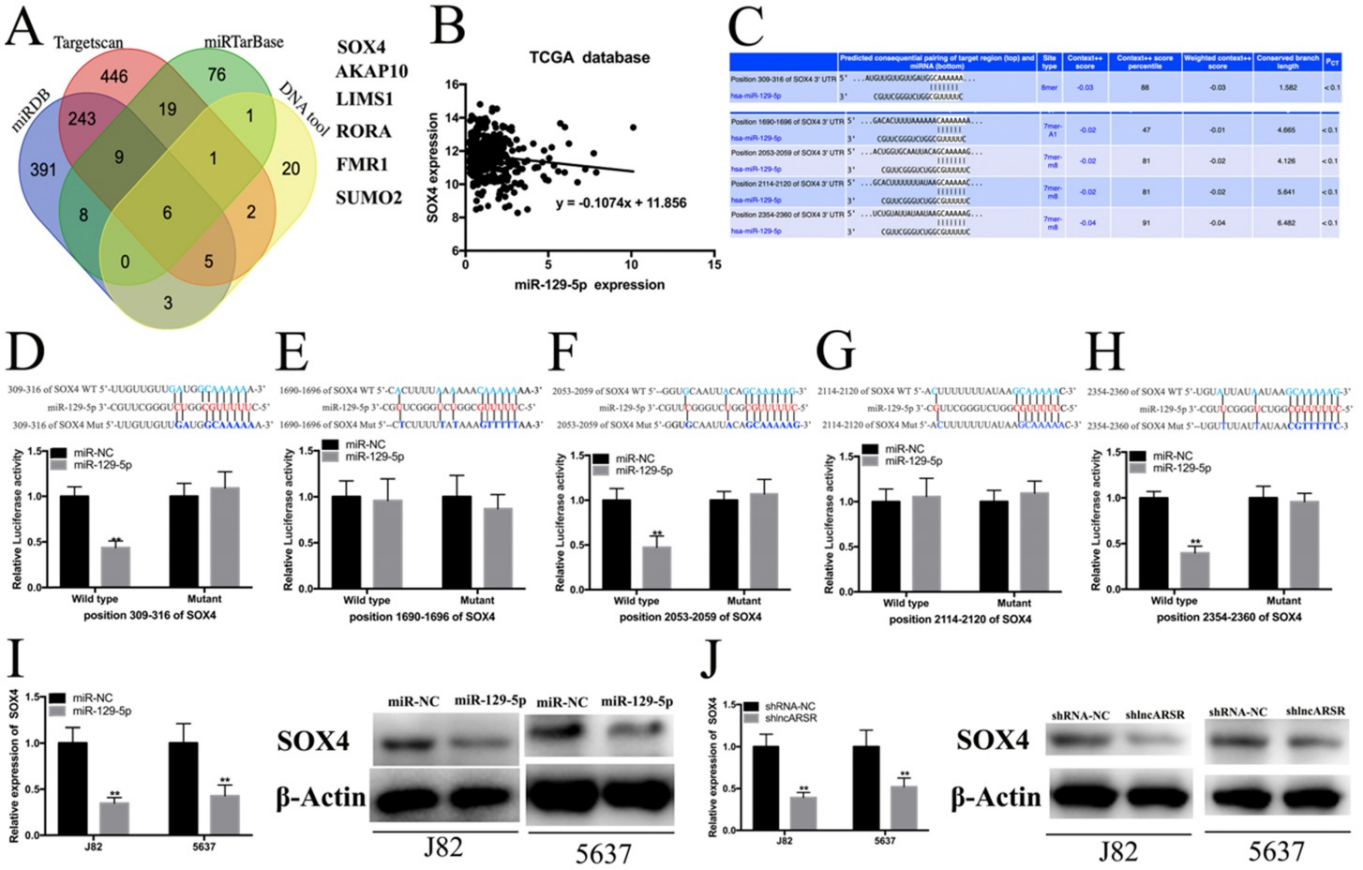
** miR-129-5p directly target SOX4. (A)** Six candidate genes were obtained by the bio-information prediction. **(B)** miR-129-5p expression were statistically positively correlated with SOX4 expression in Bca tissues from TCGA database. **(C)** Bioinformatics analysis showed that the 3'UTR sequence of SOX4 is complementary to the seed sequence of miR-129-5p. **(D-H)** Dual-luciferase reporter assay was performed to detect the putative binding sites between miR-129-5p and the 3'UTR sequence of SOX4. **(I)** Increased expression of miR-129-5p attenuated the mRNA and protein expression of SOX4 in Bca cells. **(J)** Knockdown of miR-129-5p suppressed the mRNA and protein expression of SOX4 in Bca cells.

To investigate the mechanism of miR-129-5p suppressing the progress of Bca at the molecular level, we combined the miRDB, Targetscan, miRTarBase and DNA tool database to predict the target genes of miR-129-5p. Six candidate genes in total were obtained, including SOX4, AKAP10, LIMS1, RORA, FMR1, SUMO2 (Figure. 6A). Subsequently, we performed the comprehensive transcriptional analysis by extracting the data from TCGA dataset. The results showed that miR-129-5p expression was statistically negatively corelated with SOX4 expression in Bca tissues (Figure. 6B). Further bio-information analysis suggested that SOX4 had five putative binding sites with miR-129-5p (Figure. 6C). Dual-luciferase reporter assay showed that SOX4 had three verified binding with miR-129-5p, including the position of 309-316 bp, 2053-2059 bp and 2354-2360 bp of SOX4 3′-UTR, whereas co-transfection of the other position of SOX4 3′-UTR WT and miR-129-5p showed no remarkable change in luciferase activity, including the position of 1690-1696 bp and 2114-2120 bp (Figure. 6D-H). Moreover, overexpression of miR-129-5p decreased the mRNA and protein expression of SOX4 in Bca cells (Figure. 6I). Knockdown of lncARSR could also suppress the SOX4 mRNA and protein expression (Figure. 6J).

### Silence of lncARSR inhibits the growth of Bca cells *in vivo*

**Figure 7:**
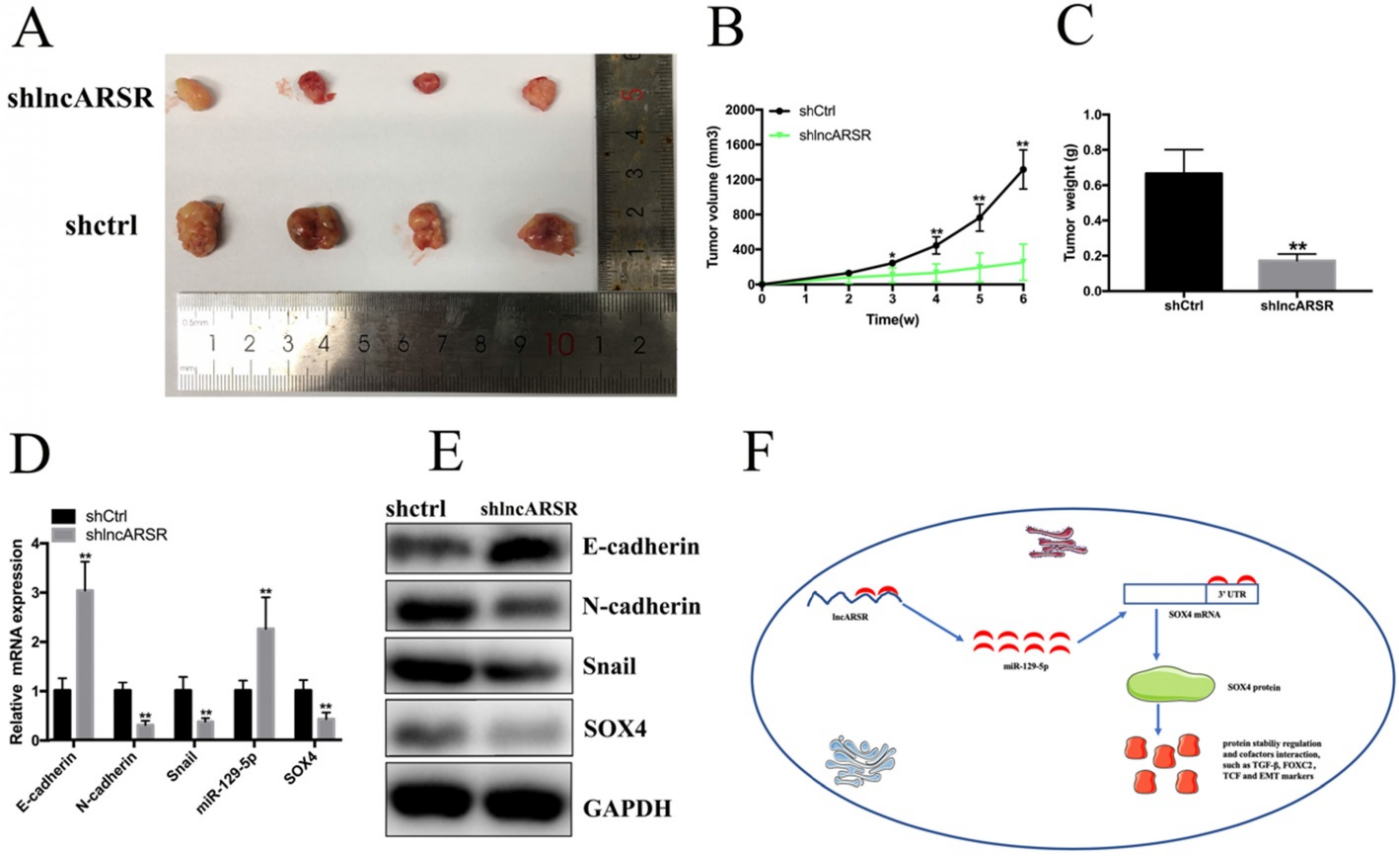
** Knockdown of lncARSR inhibits tumorigenesis of Bca cells *in vivo*. (A)** Representative images of xenograft Bca tumors. **(B)** Tumor volume curve of xenograft tumors were measured every week. **(C)** Tumor weight was weighted when the mice were euthanized. **(D** and** E)** Suppression of lncARSR inhibited EMT process, increased miR-129-5p and decreased SOX4 expression *in vivo*. **(F)** The schematic diagram of the mechanism of lncARSR/miR-129-5p/SOX4 axis in Bca.

To detect the functions of lncARSR *in vivo*, 5637 cells transfected with shlncARSR or shCtrl was injected into the nude mice to establish xenograft tumor model (Figure. 7A). The tumors derived from the lncARSR-deficient cells were much small in size than that from the negative group (Figure. 7B). Tumor weight of shlncARSR group was less than the shCtrl group (Figure. 7C). In addition, knockdown of lncARSR inhibited the expression of SOX4 and EMT process *in vivo* (Figure. 7D, E). These results indicated that lncARSR promoted tumorigenicity of Bca cells through increasing SOX4 expression.

## Discussion

LncRNAs are generally defined as transcripts of more than 200 nucleotides without evident protein coding potential which regulate gene expression related to cell proliferation, migration, invasion, apoptosis and drug resistance at different levels in multiple tumors 21, 22. They can interact with DNA, RNA or protein molecules to regulate gene expression and exert cellular effects via modulating different mechanisms 23. Dysregulation of lncRNAs may cause internal environment disturbance and sickness, especially cancers. LncRNA SPRY4-IT1 has been reported to inhibit the expression of miR-101-3p and subsequently increase the expression of EZH2 in a ceRNA-dependent manner to promote proliferation and metastasis of Bca cells 24. LncRNA BLACAT2 can promote bladder cancer-associated lymphangiogenesis and lymphatic metastasis both *in vitro* and *in vivo* 25. Hence, it is urgent to get deep insight into the lncRNAs associated mechanisms to reveal new and unanticipated biology to advance our understanding of cancer treatment.

LncARSR, a recently identified lncRNA with 591 nucleotides in length, playing important role in cancer development. In renal cancer, lncARSR functions as miRNA sponge to competitively bind with miR-34 and miR-449, which can up-regulate AXL/c-MET and activate STAT3, AKT, and ERK signaling 18. In addition, lncARSR can also promote the self-renewal capacity, tumorigenicity and metastasis of renal tumor initiating cells thorough interacting with Yes-associated protein (YAP) to block its phosphorylation by LATS1 26. In hepatocellular carcinoma, lncARSR promotes doxorubicin resistance via regulating PTEN-PI3K/Akt pathway 19. LncARSR was up-regulated in liver cancer stem cells (CSCs) and promoted hepatocellular carcinoma cells dedifferentiation and liver CSCs separation by modulating STAT3 signaling 27. However, the clinical significance and regulatory role in Bca remains unknown.

In this study, we found that lncARSR expression was significantly higher in Bca tissues compared with matched adjacent normal tissues. Increased expression of lncARSR was correlated with high histological grade and large tumor size in Bca patients. Bca patients with high lncARSR expression have a reduced overall survival time and disease-free survival time. Further experiments demonstrated that knockdown of lncARSR inhibited the proliferation, migration and invasion of Bca cells. Mechanistically, lncARSR was predominately located in the cell cytoplasm, indicating that lncARSR may play a role in posttranscriptional level and act as a nature miRNA sponge. Importantly, bioinformatics databases assay and RNA pull down assay proved that lncARSR could competitively bind with miR-129-5p in Bca cells. Knockdown of lncARSR could increase the expression of miR-129-5p. Taken together, our results revealed that lncARSR could act as an effective miRNAs sponge. Further rescue assay demonstrated that overexpression of miR-129-5p reversed the malignant phenotype inhibition of Bca cells induced by silencing lncARSR, such as proliferation, migration and invasion. In addition, we found that miR-129-5p expression was negatively corelated with SOX4 expression in Bca tissues from TCGA database and overexpression of miR-129-5p decreased SOX4 expression in Bca cells. Besides, knockdown of lncARSR can inhibit the mRNA and protein expression of SOX4 in Bca cells. Recently, aberrant Sox4 expression has been observed in various cancer types, including breast, bladder, glioblastoma, liver and colorectal, and associates with poor prognosis and disease progression 28-31. SOX4 is a crucial regulator in maintaining the mesenchymal state of EMT process. Aberrant expression of SOX4 in epithelial cells from various tissues cause the induction of a mesenchymal phenotype corelated with increased expression of N-cadherin, vimentin and fibronectin and decreased expression of E-cadherin, as well as enhanced cell migration and invasion. Indeed, SOX4 is directly involved in the induction of mesenchymal markers associated with cell proliferation, migration, and metastasis 32, 33. A meta-analysis showed that overexpression of SOX4 was positively associated with poorer overall survival 34. Based on these biological functions, SOX4 can regulate the proliferation, migration, invasion and apoptosis in Bca cells. Through searching online bioinformatics database, bio-information analysis predicted that lncARSR and SOX4 have common putative binding sites with miR-129-5p. Further experiments demonstrated that knockdown of lncARSR increased miR-129-5p expression and subsequently inhibited the expression of SOX4 in a ceRNA-dependent manner.

## Conclusions

Our results suggested that lncARSR acts as a nature miRNA sponge to positively regulate SOX4 expression through sponging miR-129-5p and subsequently promotes the proliferation, migration and invasion of Bca cells, thus playing an oncogenic role in the progression of bladder cancer. Our study provided a new basis for investigating the development and progression of bladder cancer. In general, our data reveals that lncARSR is a powerful tumor biomarker for the diagnosis and treatment of Bca.

## Supplementary Material

Supplementary table 1.

## Figures and Tables

**Table 1:** Correlation between lncARSR expression levels and clinicopathological features of Bca patients.

Parameters	Group	Total	lncARSR expression		P value
			high low		
Gender	male	40	23	17	0.235
	female	22	16	6	
Age	< 60	18	10	8	0.444
	≥ 60	44	29	15	
Histological grade	low	26	9	17	0.000^*^
	high	36	30	6	
Tumor size	< 3 cm	23	9	14	0.011^*^
	≥ 3 cm	39	28	11	
Tumor stage	Ta, T1	10	5	5	0.356
	T2-T4	52	34	18	
Lymph nodes metastasis	No	43	29	14	0.266
	Yes	19	10	9	
Distant metastasis	M0	56	36	20	0.491
	M1	6	3	3	

*P < 0.05 was considered significant (Chi-square test between 2 groups).
