# Supplementary material for: LncARSR sponges miR-129-5p to promote proliferation and metastasis of bladder cancer cells through increasing SOX4 expression
Source: Int J Biol Sci. 2020 Jan 1;16(1):1–11. doi: 10.7150/ijbs.39461 (PMC6930381; doi:10.7150/ijbs.39461)
Supplement: Supplementary file 1 — Supplementary table 1. [file ijbsv16p0001s1.pdf]

Supplementary Table 1. The primer sequences included in this study.

Gene		Primer sequences (5'-3')
lncARSR	Forward	TTTGAAATGCTCTTTGAGGGAT
	Reverse	TGCAGGTTGTCTGAAGTTGGA
E-cadherin	Forward	ATTTTTCCCTCGACACCCGAT
	Reverse	TCCCAGGCGTAGACCAAGA
N-cadherin	Forward	AGCCAACCTTAACTGAGGAGT
	Reverse	GGCAAGTTGATTGGAGGGATG
Snail	Forward	CGAGTGGTTCTTCTGCGCTA
	Reverse	GGGCTGCTGGAAGGTAAACT
SOX4	Forward	CGAGTGGTTCTTCTGCGCTA
	Reverse	GGGCTGCTGGAAGGTAAACT
GAPDH	Forward	ACACCATGGGGAAGGTGAAG
	Reverse	AAGGGGTCATTGATGGCAAC
U6	Forward	CTCGCTTCGGCAGCACA
	Reverse	AACGCTTCACGAATTTGCGT
miR-346	Forward	AGTGTCTGCCCGCAT
miR-3168	Forward	CGCAGGAGTTCTACAGTCA
miR-6817-5p	Forward	GTCTGCCATAGGAAGCTTG
miR-4769-3p	Forward	GCAGTCTGCCATCCTC
miR-129-5p	Forward	CAGCTTTTTGCGGTCTG
miR-142-3p	Forward	CGCAGTGTAGTGTTTCCT
miR-499b-5p	Forward	CAGACAGACTTGCTGTGATG
miR-378a-5p	Forward	TCCTGACTCCAGGTCCT
